# B-Cell ST6Gal1/Neuraminidase 1 Ratios Inversely Predict the Combined Remission and Low-Disease-Activity Subgroup with DAS28-MCP-1 and SDAI Scores for Rheumatoid Arthritis

**DOI:** 10.3390/ijms26178226

**Published:** 2025-08-25

**Authors:** Lieh-Bang Liou, Ping-Han Tsai, Yao-Fan Fang, Yen-Fu Chen, Che-Tzu Chang, Chih-Chieh Chen, Wen-Yu Chiang

**Affiliations:** 1 Division of Rheumatology, Allergy and Immunology, New Taipei Municipal Tucheng Hospital, New Taipei 236017, Taiwan; a9232@cgmh.org.tw (P.-H.T.); pisces4018@cgmh.org.tw (C.-C.C.); lugia7788@cgmh.org.tw (W.-Y.C.); 2 Division of Rheumatology, Allergy and Immunology, Chang Gung Memorial Hospital at Linkou, Taoyuan 333423, Taiwan; 8802012@cgmh.org.tw (Y.-F.F.); patrichen0693@gmail.com (Y.-F.C.); chang3109@cgmh.org.tw (C.-T.C.); 3 School of Medicine, Chang Gung University College of Medicine, Taoyuan 333323, Taiwan

**Keywords:** DAS28 scores, Neu1, sialic acid, ST6Gal1, 2005 ARA remission, 2011 ACR/EULAR remission

## Abstract

The associations between sialylated anti-cyclic citrullinated peptide (anti-CCP) antibodies bearing α-2,6-sialic acid (SIA), ST6Gal1 and Neu1 enzymes, and clinical disease activity measures such as disease activity score 28 (DAS28), the Simplified Disease Activity Index (SDAI), and Clinical Disease Activity Index (CDAI) are unknown in rheumatoid arthritis (RA). To address this gap, this study included 97 patients with RA evaluated at baseline (month 0) and at 6 and 12 months. At each visit, blood cells were analyzed for B-cell ST6Gal1 and Neu1 expressions, and plasma samples were assessed for ST6Gal1 and Neu1 levels. The erythrocyte sedimentation rate (ESR), C-reactive protein (CRP), monocyte chemotactic protein-1 (MCP-1), and IgG anti-CCP with its α-2,6-SIA modification were measured. Disease activity measures, namely DAS28-ESR, DAS28-CRP, DAS28-MCP-1, SDAI, and CDAI, were calculated. Correlations and Receiver Operating Characteristics among ST6Gal, Neu1, SIA/anti-CCP ratios, and disease activity measures were assessed. Multivariate regression analyses were performed to reveal confounding factors in such correlations. The total SIA content of anti-CCP antibodies was inversely correlated with B-cell Neu1 levels (ρ = −0.317 with *p* = 0.013. Plasma (free-form) Neu1 levels were inversely correlated with SIA/IgG anti-CCP ratios (ρ = −0.361, *p* = 0.001) in the DAS28-MCP-1 < 2.2 (remission) subgroup. No such correlation was observed for the DAS28-ESR, DAS28-CRP, SDAI, or CDAI subgroups. B-cell ST6Gal1 levels correlated inversely with SDAI ≤ 11 and DAS28-MCP-1 ≤ 3.6 combined remission and low-disease-activity subgroups (ρ = −0.315 with *p* = 0.001 and ρ = −0.237 with *p* = 0.008, respectively). The same was observed for B-cell ST6Gal1/Neu1 ratios correlating with the SDAI ≤ 11 subgroup (ρ = −0.261, *p* = 0.009). Nevertheless, B-cell ST6Gal1/Neu1 ratios against SDAI ≤ 11 and DAS28-MCP-1 ≤ 3.6 subgroups produced significant area-under-curve (AUC) values of 0.616 and 0.600, respectively (asymptotic *p*-Values 0.004 and 0.018, respectively). Through multivariate regression analyses, we found that biologics (a confounding factor) interfered with *p*-Values related to the B-cell ST6Gal1 enzyme but did not interfere with *p*-Values related to the pure B-cell Neu1 enzyme. In addition, disease duration interfered with *p*-Values related to the pure Neu1 enzyme on B-cells or in plasma. Moreover, plasma ST6Gal1/Neu1 ratios against the DAS28-MCP-1 < 2.2 remission subgroup produced an AUC of 0.628 and asymptotic *p* = 0.003. Therefore, it is suggested that B-cell ST6Gal1/Neu1 ratios can be used as clinical indicators for the combined remission and low-disease-activity subgroup of SDAI and DAS28-MCP-1 formulae. Plasma ST6Gal1/Neu1 ratios are also good indicators of DAS28-MCP-1 remission.

## 1. Introduction

Sialyltransferases (STs) are enzymes that catalyze the transfer of sialic acid (SIA) to nascent glycoproteins or glycolipids [1,2]. Among STs, α-2,6-ST I (ST6Gal1) catalyzes the transfer of an α-2,6-linked SIA residue to the terminal galactose of type 2 disaccharides (Gal α1-4GlcNAc). ST6Gal1 is localized to the cell membrane, the Golgi apparatus, and the extracellular space [2]. It also contributes to the sialylation of IgG Fc regions through modifications involving α1-3 mannose residues [3]. Notably, elevated ST activity has been reported in B cells from patients with primary Sjögren’s syndrome [4].

In contrast to STs, neuraminidase (Neu) enzymes remove SIA from cells. Among these enzymes, Neu1 is essential for early IL-4 production by T cells during their interaction with antigen-presenting cells [5], the regulation of macrophage phagocytosis [6], and the production of IgG1 and IgE by B cells [7]. Neu1 is typically localized at both the cell surface and in the lysosomal compartment [8], demonstrating its involvement in both extracellular and intracellular processes.

To summarize, ST6Gal1 and Neu1 operate within the α-2,6-SIA regulatory pathway. Notably, B-cell Neu1 levels are strong predictors of remission as defined by two stringent remission criteria for rheumatoid arthritis (RA) [9,10,11], whereas B-cell ST6Gal1 levels are associated only with remission status, as defined by the 2005 modified American Rheumatism Association (ARA) criteria [11].

IgG anti-cyclic citrullinated peptide (anti-CCP) antibodies have been used for over a decade to predict bone erosion in RA. However, their association with either RA disease activity measures or the remission criteria remains undocumented. Sialylated anti-collagen II and anti-citrullinated protein antibodies suppress the onset and severity of collagen-induced arthritis in mice [12]; hence, investigating whether sialylated IgG anti-CCP antibodies are indicators of disease activity or true remission in patients with RA can provide valuable insights. Accordingly, we previously demonstrated that the ratios of α-2,6-SIA concentrations to sialylated IgG anti-CCP antibody concentrations (SIA/anti-CCP ratios) can predict remission based on disease activity 28-erythrocyte sedimentation rate (DAS28-ESR) scores, with DAS28-ESR scores of <2.6 indicating remission and DAS28-ESR scores of ≥2.6 indicating active disease [13].

The DAS28-ESR-based remission criterion is not consistent with the two aforementioned remission criteria as the corresponding concordance rates are only 36.99% and 49.13%, respectively [14]. By contrast, the newly developed DAS28-MCP-1 score demonstrates higher concordance with the two aforementioned remission criteria (75.61% and 81.71%, respectively) [14]. However, studies have yet to examine whether SIA/anti-CCP ratios align with the two RA remission definitions [9,10] and the DAS28-MCP-1 remission criterion (<2.2). Specifically, it is currently unknown whether SIA/anti-CCP ratios are correlated with B-cell Neu1 and ST6Gal1 levels and whether they effectively align with the two remission definitions and DAS28-MCP-1 criterion [14] in patients with RA.

ST6Gal1 was identified in plasma and was responsible for the α-2,6-sialylation of IgG [15]. However, other research suggests that ST6Gal1 is not essential for this modification [16]. Similarly, Neu1 was present in the bloodstream [17]. These conflicting findings motivated us to investigate plasma ST6Gal1 and Neu1 levels and examine their correlations with SIA/anti-CCP ratios, various disease activity measures, and the two established remission definitions. Accordingly, we examined the correlations between the biomarkers SIA/anti-CCP ratios, B-cell Neu1, B-cell ST6Gal1, plasma ST6Gal1, and plasma Neu1 and their connection to various disease activity subgroups and the two remission definitions in patients with RA.

## 2. Results

### 2.1. Baseline Characteristics

We recruited 97 patients with RA. Table 1 presents the patients’ demographic and clinical/laboratory data. The mean age was 57.5 ± 10.6 years (mean ± standard deviation). The tender joint count (TJC) was 3.1 ± 3.4 (range: 0–15), and the swollen joint count (SJC) was 2.1 ± 2.7 (range: 0–11), indicating a broad spectrum of disease activity, from minimal to high. Both rheumatoid factors (RFs) and IgG anti-CCP antibody titers were markedly high.

### 2.2. Correlations Betweeen SIA/Anti-CCP Ratios, B-Cell SIA-Related Enzymes, and DAS28 Score

**Figure 1:**
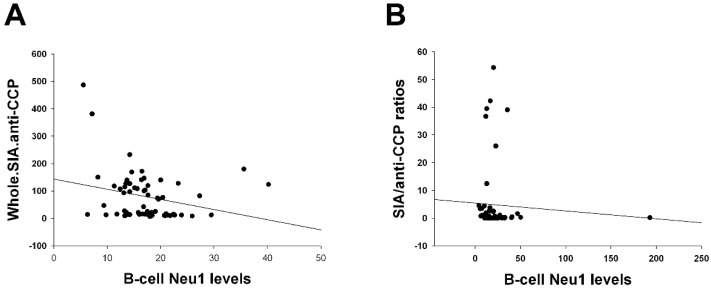
The correlation between sialic acid (SIA)/IgG anti-CCP antibodies and B-cell neuraminidase 1 (Neu1) levels. (**A**) Whole SIA amounts of IgG anti-CCP antibodies (Whole.SIA.anti-CCP) were inversely correlated after 12 months with B-cell Neu1 levels (*n* = 61; ρ = −0.317, *p* = 0.013). The *p*-Value is statistically significant and lower than 0.017 (0.05 divided by three correlations). (**B**) SIA/IgG anti-CCP (SIA/anti-CCP) ratios, pooled from 0, 6, and 12 month patients with combined remission and low disease activity score 28 through monocyte chemotactic protein-1 (DAS28-MCP-1) ≤ 3.6, also exhibited an inverse correlation with B-cell Neu1 levels (*n* = 62; ρ = −0.253, *p* = 0.047). The *p*-Value is statistically non-significant and higher than a significant *p*-Value = 0.017 (0.05 divided by three correlations).

As expected, the total SIA content of IgG anti-CCP antibodies and the SIA/anti-CCP ratio (the latter was tested in the DAS28-MCP-1 <3.6 subgroup, though results were statistically non-significant after Bonferroni correction) were significantly and inversely correlated with B-cell Neu1 levels (Figure 1A,B). Multivariate regression analyses were performed using statistically significant correlations, as presented in the Section 3.

**Figure 2:**
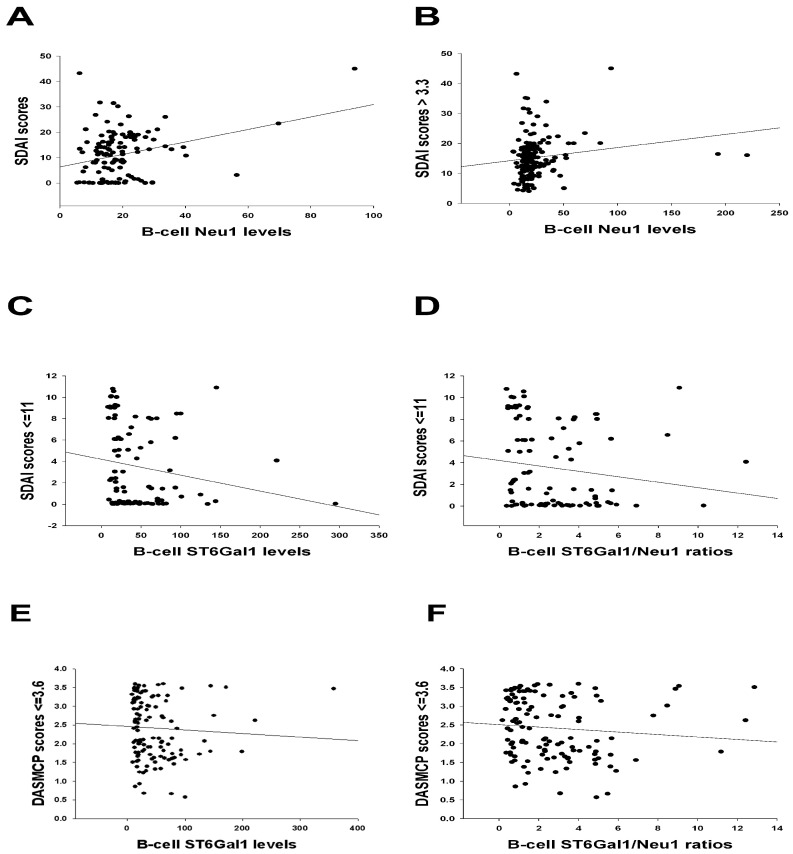
The correlation between B-cell SIA-related enzyme levels/ratios and the simplified disease activity score (SDAI) scores and DAS28-MCP-1 scores. (**A**) B-cell Neu1 levels were positively correlated with SDAI scores at 6 months (*n* = 65; ρ = 0.203, *p* = 0.023). The *p*-Value is statistically non-significant and higher than 0.017 (0.05 divided by three correlations). (**B**) B-cell Neu1 levels were positively correlated with non-remission SDAI scores > 3.3 (*n* = 122; ρ = 0.228, *p* = 0.005). (**C**) B-cell ST6Gal1 levels were inversely correlated with combined remission and low disease activity SDAI scores ≤ 11 (*n* = 99; ρ = −0.315, *p* = 0.001). (**D**) B-cell ST6Gal1/Neu1 ratios were inversely correlated with combined remission and low disease activity SDAI scores ≤ 11 (*n* = 99; ρ = −0.261, *p* = 0.009). The *p*-Values in (**B**–**D**) are statistically significant and lower than 0.017 (0.05 divided by three correlations). (**E**) B-cell ST6Gal1 levels were inversely correlated with the combined remission and low disease activity DAS28-MCP-1 scores ≤ 3.6 (*n* = 124; ρ = −0.237, *p* = 0.008). The *p*-Value is statistically significant and lower than 0.017 (0.05 divided by three correlations). (**F**) B-cell ST6Gal1/Neu1 ratios were inversely correlated with combined remission and low disease activity DAS28-MCP-1 scores ≤ 3.6 (*n* = 124; ρ = −0.203, *p* = 0.024). The *p*-Value is statistically non-significant and higher than 0.017 (0.05 divided by three correlations).

B-cell Neu1 levels were positively correlated with SDAI scores at 6 months (as shown in Figure 2A, though this was statistically non-significant after Bonferroni correction) and with SDAI scores above the non-remission threshold (>3.3; Figure 2B), suggesting that elevated B-cell Neu1 levels are an indicator of higher disease activity. Conversely, B-cell ST6Gal1 levels and ST6Gal1/Neu 1 ratios were inversely correlated with SDAI scores ≤ 11 (remission and low disease activity; Figure 2C,D). These findings suggest that higher B-cell ST6Gal1 levels and ST6Gal1/Neu 1 ratios are associated with lower SDAI scores, aligning with clinical expectations. Similarly, B-cell ST6Gal1 levels and ST6Gal1/Neu 1 ratios were inversely correlated with DAS28-MCP-1 scores of ≤3.6 (remission and low disease activity, as shown in Figure 2E,F, though2F was statistically non-significant after Bonferroni correction). Moreover, B-cell ST6Gal1 levels were significantly higher in the DAS28-MCP-1 < 2.2 subgroup than in the DAS28-MCP-1 ≥ 2.2 subgroup (*p* = 0.01, using Mann–Whitney U test). Hence, high B-cell ST6Gal1 levels and ST6Gal1/Neu 1 ratios indicate low DAS28-MCP-1 scores.

To further assess the enzyme biomarkers in Figure 2 to predict disease activity score subgroups, we formulated Receiver Operating Characteristic (ROC) curves for the SDAI and DAS28-MCP-1 subgroups (Table 2). It is evident that B-cell ST6Gal1/Neu1 ratios against both SDAI < 11 and DAS28-MCP-1 ≤ 3.6 subgroups (both of which belong to the combined remission and lower disease activity subgroup) rendered a significant AUC ≥ 0.600.

B-cell ST6Gal1/Neu1 ratios were inversely correlated with five disease activity scores (DAS28-ESR, DAS28-CRP, DAS28-MCP-1, SDAI, and CDAI). Among these, only the correlations with DAS28-ESR reached statistical significance (values lower than 0.01 were adjusted by *p*-Value after Bonferroni correction was performed with 0.05 values divided by five correlations) and acceptable coefficients (lower than −0.02) (Table 3).

### 2.3. Correlations Between Different B-Cell SIA-Related Enzymes

Across different time points and various DAS28 and SDAI measures, B-cell Neu1 was inversely correlated with B-cell ST6Gal1/Neu1 ratios, whereas B-cell ST6Gal1 levels were positively correlated with B-cell ST6Gal1/Neu1 ratios (Supplementary Table S1). Notably, among CDAI categories, B-cell Neu1 and B-cell ST6Gal1 were consistently inversely and positively correlated with B-cell ST6Gal1/Neu1 ratios, respectively (Supplementary Table S2).

### 2.4. Correlation Between SIA/Anti-CCP Ratios and Plasma Neu1 Levels Across Different Disease Activity, Remission, and Non-Remission Categories

To expand on the results presented in Figure 1, we further examined (free-form) plasma Neu1 levels and their correlation with SIA/anti-CCP ratios (Supplementary Figure S1). SIA/anti-CCP ratios were inversely correlated with plasma Neu1 levels in patients with DAS28-MCP-1 scores of <2.2, but not in those with scores ≥ 2.2 (Supplementary Figure S1A,B). Similarly, an inverse correlation was observed in patients with CDAI scores ≤ 2.8 (however, this was statistically non-significant after Bonferroni correction), but not in those with scores > 2.8 (Supplementary Figure S1C,D). No such correlation was observed in the DAS28-ESR, DAS28-CRP, or SDAI subgroups.

We further examined the correlations between (free-form) plasma Neu1 levels and SIA/anti-CCP ratios in terms of the remission criteria defined by the modified definitions of the 2005 ARA and the 2011 American College of Rheumatology/European League Against Rheumatism (ACR/EULAR) [9,10]. Notably, SIA/anti-CCP ratios were inversely correlated with plasma Neu1 levels in patients who met the 2005 modified ARA remission definition (though this was statistically non-significant after Bonferroni correction), but not in those who did not (Supplementary Figure S2A,B). A similar inverse correlation was observed in patients who met the 2011 ACR/EULAR remission definition (though this was statistically non-significant after Bonferroni correction), but not in those who did not (Supplementary Figure S2C,D). Interestingly, plasma ST6Gal1 levels were significantly higher (*p* = 0.012) in patients who met the 2005 modified ARA remission definition (*n* = 48) than in those who did not (*n* = 168).

**Figure 3:**
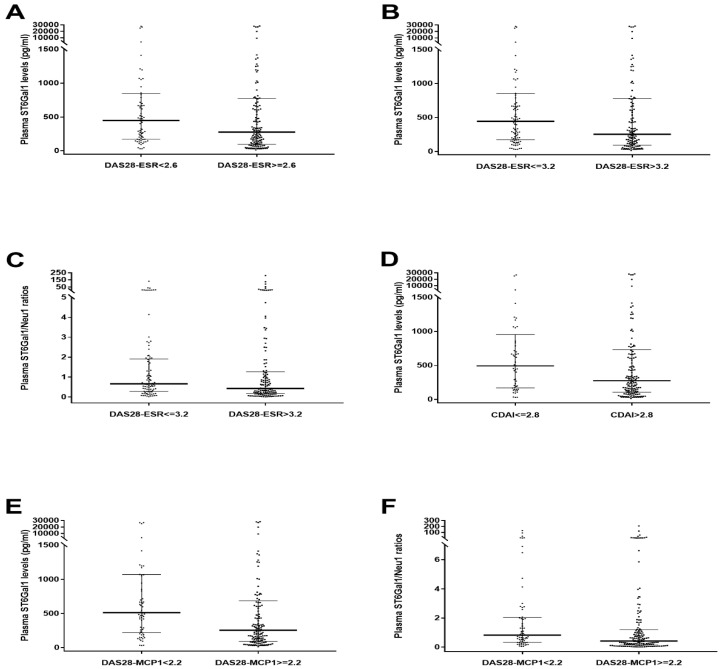
Comparison of (free-form) plasma ST6Gal1 levels and ST6Gal1/Neu1 ratios across disease activity subgroups. (**A**) Plasma ST6Gal1 levels were significantly higher in the DAS28-ESR < 2.6 (remission) subgroup (*n* = 73) than in the DAS28-ESR ≥ 2.6 (non-remission) subgroup (*n* = 153; *p* = 0.046). (**B**) Plasma ST6Gal1 levels were significantly higher in the DAS28-ESR ≤ 3.2 (remission and low activity) subgroup (*n* = 96) than those in the DAS28-ESR > 3.2 (moderate and high activity) subgroup (*n* = 130; *p* = 0.024). (**C**) Plasma ST6Gal1/Neu1 ratios were higher in the DAS28-ESR ≤ 3.2 (remission and low activity) subgroup (*n* = 96) than those in the DAS28-ESR > 3.2 (moderate and high activity) subgroup (*n* = 130; *p* = 0.039). (**D**) Plasma ST6Gal1 levels were higher in the CDAI ≤ 2.8 (remission) subgroup (*n* = 53) than those in the CDAI > 2.8 (non-remission) subgroup (*n* = 166; *p* = 0.041). (**E**) Plasma ST6Gal1 levels were higher in the DAS28-MCP-1 < 2.2 (remission) subgroup (*n* = 64) than those in the DAS28-MCP-1 ≥ 2.2 (non-remission) subgroup (*n* = 153; *p* = 0.001). (**F**) Plasma ST6Gal1/Neu1 ratios were higher in the DAS28-MCP-1 < 2.2 (remission) subgroup (*n* = 64) than those in the DAS28-MCP-1 ≥ 2.2 (non-remission) subgroup (*n* = 153; *p* = 0.003). All comparisons were conducted using the Mann–Whitney U test. All *p*-Values were less than 0.05 according to Bonferroni correction (one comparison was performed for each figure).

We also investigated whether enzyme levels and ratios differed across disease activity categories for plasma enzymes. Plasma ST6Gal1 levels were significantly higher in remission or combined remission and low-activity subgroups defined by DAS28-ESR < 2.6, DAS28-ESR ≤ 3.2, CDAI ≤ 2.8, and DAS28-MCP-1 < 2.2 than in their respective non-remission or moderate-to-high-activity counterparts (Figure 3A,B,D,E). Similarly, plasma ST6Gal1/Neu1 ratios were significantly elevated in DAS28-ESR ≤ 3.2 (combined remission and low-activity) and DAS28-MCP-1 < 2.2 (remission) subgroups compared with combined moderate- and high-activity and non-remission subgroups, respectively (Figure 3C,F). These differences were not observed in the DAS28-CRP or SDAI subgroups.

Further ROC analysis revealed that plasma ST6Gal1 levels against DAS28-MCP-1 < 2.2 and modified 2005 ARA remission provided an AUC > 0.600 with significant *p*-Values (Table 4). In addition, plasma ST6Gal1/Neu1 ratios indicated a meaningful AUC in the DAS28-MCP-1 < 2.2 remission subgroup (Table 4).

## 3. Discussion

Both serum and synovial fluid anti-citrullinated peptide antibodies (ACPAs) lack terminal sialic acid residues for their Fc fragments in patients with RA [18]. The effect of re-sialylation on arthritogenic IgG has been shown to inhibit collagen-induced arthritis in mice [12]. Moreover, the sialylation of ACPA is inversely correlated with inflammatory indicators (CRP, ESR) and is negatively associated with ACPA–immune complex-induced tumor necrosis factor α (TNFα) production [19]. Then, TNFα induces an inflammatory reaction in RA. Both ST6Gal1 and Neu1 levels are involved in the α-2,6-sialylation of sialic acid. The correlation of these two enzymes with the α-2,6-sialic acid of autoantibodies has not been demonstrated in clinical patients until now.

ST6Gal1 is involved in the Fc sialylation of IgG and provides an anti-inflammation role of IgG antibodies [3]. Neu1 has been demonstrated to involve in T- and B-cell, and macrophage function [5,6,7]. Hence, both ST6Gal1 and Neu 1 enzymes certainly hold a significant role in the immune-inflammatory reaction. One recent paper demonstrated that the B-cell Neu1 level indicates strongly the 2005 modified ARA remission and the ACR/EULAR remission [11]. Whereas, the B-cell ST6Gal1 level is connected with the 2005 modified ARA remission only [11]. Both B-cell ST6Gal1 and Neu1 levels discriminate the improvement subgroup and the non-improvement subgroup, as defined by ACR, EULAR, and SDAI criteria [11]. Nevertheless, there is still no publication to show the connection between ST6Gal1, Neu 1, or ST6Gal1/Neu1 ratios and disease activity of rheumatoid arthritis until now. In this study, we firstly uncover that B-cell ST6Gal1/Neu1 ratios inversely forecast the combined remission and low-disease-activity subgroup with DAS28-MCP-12 ≤ 3.6 and SDAI ≤ 11 scores for rheumatoid arthritis (Table 2). Moreover, plasma ST6Gal1/Neu1 ratios also indicate inversely the remission DAS28-MCP-1 < 2.2 subgroup (Table 4). Therefore, the ST6Gal1/Neu1 ratio is a potential predictor for remission or combined remission and low disease activity for DAS28-MCP-1 and SDAI assessment in rheumatoid arthritis, pending confirmation by other studies. Nevertheless, the usefulness not applied to DAS28-CRP and CDAI scores may be a major barrier for a widespread clinical usage at this time. A future study employing longitudinal modeling for repeated measures with complete variable data may provide insight into cause-and-effect relationships. Moreover, how to comprehend both the B-cell and plasma ST6Gal1/Neu1 ratios into a single formula is challenging and worth pursuing in the future.

This is the first study to report a significant inverse correlation between SIA/IgG anti-CCP ratios and B-cell Neu1 levels (Figure 1A) and plasma Neu1 levels (Supplementary Figure S1A). In particular, B-cell Neu1 levels were inversely correlated with B-cell ST6Gal1/Neu1 ratios across different follow-up time points and various DAS28 score categories (Supplementary Table S1), indicating their opposing dynamics. Furthermore, B-cell Neu1 and ST6Gal1 levels, as well as ST6Gal1/Neu1 ratios, were positively or negatively correlated with various SDAI score categories (Figure 2), indicating their involvement in SDAI-related disease activity. Additionally, B-cell ST6Gal1 levels and ST6Gal1/Neu1 ratios were correlated with low disease activity or remission in patients with DAS28-MCP-1 scores ≤ 3.6 (Figure 2), implying their role in modulating low DAS28-MCP-1 activity. Overall, these findings demonstrate that B-cell Neu1 and ST6Gal1 levels, as well as the ST6Gal1/Neu1 ratio, may contribute to the progression and regulation of disease activity in RA.

Moreover, this study identified that plasma ST6Gal1 levels predict the DAS28-MCP-1 < 2.2 remission subgroup and modified 2005 ARA Remission (Table 4) while plasma ST6Gal1/Neu1 ratios predict the DAS28-MCP-1 < 2.2 subgroup (Table 4). Therefore, apart from B-cell enzymes, the plasma detection of the ST6Gal enzyme and ST6Gal1/Neu1 ratios offers a new and clinically useful indicator for DAS28-MCP-1 remission.

IgG sialylation has been shown to occur independently of B-cell regulation [15]; instead, it is mediated by circulating factors such as secreted hepatic ST6Gal1 and a platelet-derived nucleotide sugar donor, namely cytidine monophosphate-sialic acid [15]. However, no such report on Neu1 in patients’ circulation is available. In our analysis of plasma ST6Gal1 and Neu1 levels, ST6Gal1 can differentiate between the disease activity categories defined by DAS28-ESR, CDAI, and DAS28-MCP-1 but not those defined by DAS28-CRP and SDAI (Figure 3). Moreover, SIA/anti-CCP ratios were inversely associated with plasma Neu 1 levels only in the remission categories with DAS28-MCP-1 scores (Supplementary Figure S1) but not in those defined by DAS28-ESR, DAS28-CRP, SDAI, and CDAI. This pattern was not observed in the categories defined by the modified 2005 ARA remission and the 2011 ACR/EULAR remission definitions (Supplementary Figure S2).

Multivariate regression analyses were performed for statistically significant correlations, as discussed below (Table 5). For Figure 1A, we designated the total SIA content of IgG anti-CCP antibodies as the outcome variable, and all other factors as independent variables (the order in which variables were entered is as follows: B-cell Neu1 levels (*p* = 0.045 for the correlation), age, disease duration, IgG anti-CCP, methotrexate (MTX), sulfasasalazine (sulfasa), hydroxychloroquine (HCQ), biologics, CRP, ESR, and MCP-1). MTX, sulfasa, and HCQ were the most frequently prescribed conventional disease-modifying anti-rheumatic drugs. Non-interfering independent variables (*p*-Values still < 0.05) were age, IgG anti-CCP, HCQ, biologics, CRP, and MCP-1. Interfering independent variables (*p*-Values became ≥ 0.05) were disease duration, MTX, sulfasa, and ESR. Other correlation pairs were similarly presented (Table 5). In short, biologics affected *p*-Values related to the B-cell ST6Gal1 enzyme in multivariate regression analysis, but did not interfere with the *p*-Values that correlated with the pure B-cell Neu1 enzyme. The only exception was plasma Neu1 levels (Table 5). Disease duration interfered with *p*-Values related to the pure B-cell Neu1 enzyme, either on B-cells or in plasma (Table 5). The mechanisms behind the effects that biologics exert on the B-cell ST6Gal1 enzyme warrant future studies.

This study has several limitations. First, we did not find an inverse correlation between SIA/anti-CCP ratios and B-cell or plasma ST6Gal1 levels. This could be due to the established independence of IgG sialylation from B-cell-derived ST6Gal1 [15] and the dispensability of plasma ST6Gal1 for IgG sialylation [16]. Second, we did not observe significantly higher SIA/anti-CCP ratios in patients with DAS28-ESR remission (<2.6) compared to non-remission patients (≥2.6), which was previously reported with borderline significance (*p* = 0.061) [13]. This may reflect the mechanisms noted in the first limitation [15,16]. Third, due to the attrition of patient numbers (month 0: *n* = 96, month 6: *n* = 67, and month 12: *n* = 65), we did not perform the longitudinal modeling technique for repeated measures in this study. Fourth, a healthy control group was not included; however, we compared the remission subgroup with the non-remission subgroup as an internal control.

In summary, elevated B-cell Neu1 levels are associated with higher SDAI scores, whereas increased B-cell ST6Gal1 levels and B-cell ST6Gal1/Neu1 ratios are associated with lower SDAI scores. Similarly, higher B-cell ST6Gal1 levels and ST6Gal1/Neu 1 ratios correspond to lower DAS28-MCP-1 scores. In particular, B-cell ST6Gal1/Neu 1 ratios significantly predict the combined remission and low-disease-activity subgroup with DAS28-MCP1 and SDAI scores (Table 2). These findings are promising for future clinical practice. Our results indicate that the interaction between B-cell ST6Gal1 and Neu1 enzymes is more closely associated with the combined remission and low-disease-activity subgroup with DAS28-MCP1 and SDAI scores. The patho-clinical mechanism behind these findings will be a topic studied for future research.

## 4. Materials and Methods

### 4.1. The Recruitment of Patients with RA and Clinical Assessment

Patients who met the 1987 American College of Rheumatology criteria for RA were recruited from the Linkou branch of Chang Gung Memorial Hospital. A total of 97 patients were enrolled for the evaluation of B-cells ST6Gal1 and Neu1 and IgG anti-CCP antibody levels. Clinical and laboratory data were collected at baseline (month 0) and at 6 and 12 months. At each visit, the following measures were assessed: SJC, TJC, patient’s global assessment (PGA), evaluator’s global assessment (EGA), and the health assessment questionnaire disability index (HAQ-DI). Disease activity scores were calculated as follows: DAS28-ESR score = [0.56 × √TJC] + [0.28 × √SJC] + [0.70 × ln(ESR)] + [0.014 × PGA (visual analog scale, VAS: in mm)]; DAS28-CRP score = [0.56 ×√TJC] + [0.28 ×√SJC] + [0.36 × ln(CRP: in mg/L) + 1] + [0.014 × PGA (VAS: in mm)] + 0.96; SDAI = SJC + TJC + PGA (VAS: in cm) + EGA (VAS: in cm) + CRP (in mg/dL); CDAI = SJC + TJC + PGA (VAS: in cm) + EGA (VAS: in cm); and DAS28-MCP-1 score = [0.56 × √TJC] + [0.28 × √SJC] + [0.39 × ln(MCP-1: in pg/mL)] + [0.014 × PGA (VSA: in mm)]. These scoring methods were applied as previously described [14]. Serum IgM RF levels were examined at months 0 and 12 through nephelometry (N Latex RF Kit from Siemens Healthcare Diagnostics Products GmbH, Marburg, Germany). Remission for RA was defined as DAS28-MCP-1 < 2.2; low disease activity (LDA) was designated as 2.2 ≤ DAS28-MCP-1 ≤ 3.6; moderate disease activity was defined as 3.6 < DAS28-MCP-1 ≤ 4.8; and high disease activity was indicated by DAS28-MCP-1 > 4.8 [19]. Hence, DAS28-MCP-1 ≤ 3.6 indicates a subgroup containing RA patients in both remission and LDA.

### 4.2. Cell Staining and Flow Cytometric Analysis

Peripheral blood mononuclear cells (PBMCs) were isolated as previously described, and B-cell ST6Gal1 and Neu1 levels were assessed through flow cytometry [20]. Briefly, PBMCs suspended in phosphate-buffered saline (PBS) were stained with PE-conjugated mouse antihuman CD19 (clone HIB19; BD Pharmingen, Mountain View, CA, USA) to identify B cells. The isotype control used was PE-conjugated mouse IgG1 *k* isotype control for anti-CD19 staining (clone: MOPC-21; BD Pharmingen). Moreover, mouse monoclonal IgM anti-ST6Gal1 (clone: MOPC1041) at 0.36 µg/mL and rabbit polyclonal IgG anti-Neu1 at 0.42 µg/mL (all from Abcam, Cambridge, MA, USA) were added for each staining test. Rabbit polyclonal IgG (2 µg/mL; Jackson ImmunoResearch, West Grove, PA, USA; Sigma, St. Louis, MO, USA) was used as the control for each test. Cell yield was sometimes limited, particularly for B-cell isolation, because another concurrent study required the same RA patient samples. After centrifugation (Eppendorf 5810R, 644× *g* for 5 min) and washing, the cells were suspended in 100 µL of PBS and stained with either allophycocyanin (APC)-conjugated goat antirabbit IgG (1 µg/mL; Abcam) or APC-conjugated monoclonal rat anti-mouse IgM (clone 1B4B1, 1 µg/mL; MyBioSource). After a final wash, the cells were resuspended in 500 µL of PBS (4 °C) for analysis using the BD FACSCalibur System. A minimum of 20,000 events per sample was required. Flow cytometric data were analyzed using the FlowJo (version 7.6.1) program (FlowJo, LLC, Ashland, OR, USA).

The geometric mean fluorescence intensity for each cell depended on the enzyme level in the cell. The examined parameters were CD19, ST6Gal1, and Neu1.

### 4.3. The Measurement of Plasma ST6Gal1 and Neu1

Plasma concentrations of ST6Gal1 and Neu1 were measured using a standard sandwich ELISA protocol: First, 0.1 mL of plasma was added in duplicate to wells of a 96-well plate. Similarly, 0.1 mL of standard ST6Gal1 or Neu1 solution was added at concentrations of 0, 31.25, 62.5, 125, 250, 500, 1000, and 2000 pg/mL in duplicate. The plate was sealed and incubated for 90 min at 37 °C without agitation. It was then washed twice without immersion. Second, 0.1 mL of a biotin-labeled antibody working solution was added to each well. The plate was then sealed and incubated for 60 min at 37 °C without agitation. It was then washed three times with 1 min immersion for each wash. Third, 0.1 mL of SABC working solution was added to each well, and the plate was sealed and incubated for 30 min at 37 °C without agitation. This was followed by five washes with 1 min of immersion. Fourth, 0.09 mL of TMB substrate solution was added to each well. The plate was sealed and incubated for 10–20 min at 37 °C without agitation, accompanied by accurate TMB visualization controls. Finally, the reaction was stopped by adding 0.05 mL of a stopping solution to each well. Absorbance was measured immediately at 450 nm using an ELISA reader. Plasma concentrations of ST6Gal1 and Neu1 were determined by plotting the optical densities against the standard concentration curves for each enzyme.

### 4.4. The Determination of IgG Anti-CCP Antibodies and Their SIA Contents

Plasma samples collected at months 0, 6, and 12 were analyzed for IgG anti-CCP antibody levels and SIA content using an ELISA-based method. Specifically, ELISA plates were first coated overnight at 4 °C with a 10 μg/mL CCP peptide from MedChemExpress (Monmouth Junction, NJ, USA). After four washes with 0.05% PBST, the plates were blocked with 1% BSA (1% oxidized BSA blocking solution for SIA detection) at room temperature for 2 h. After another four washes with 0.05% PBST and 100 μL of a 1:100 dilution of purified IgG (isolated through a Protein G column, as described previously [13]) was added to each well and incubated at room temperature for 2 h. After four additional PBST washes, 100 μL of the 1:3000 dilution of antihuman IgG-HRP (Jackson Laboratory, Bar Harbor, ME, USA) was added to detect anti-CCP antibody amounts. In addition, an SNA–HRP solution at a 1:500 dilution (from EY Laboratories Inc., San Mateo, CA, USA) was used for detecting α2,6-sialylated IgG anti-CCP. The plates were incubated for 2 h at room temperature. Finally, after four final PBST washes, 100 μL of the TMB substrate was added and incubated for 10–20 min at room temperature. The reaction was stopped with 50 μL of 1 N HCl, and absorbance was measured at 450 nm using an ELISA reader (Emax^TM^ Precision Microplate Reader, Molecular Devices, Sunnyvale, CA, USA). Standard curves were generated using a serum sample with high levels of IgG-anti-CCP antibodies (504.6 units) and a relatively high α2,6-SIA content, serially diluted from 1:100 up to 1:6400, as described previously [13].

### 4.5. Statistical Analysis

After data non-normality was confirmed (using the Kolmogorov–Smirnov test), we used Spearman’s correlation to assess the correlations between SIA/anti-CCP ratios and B-cell and plasma Neu1 levels. Similarly, in Table 1, data displaying a normal distribution are shown as the mean ± S.D. and ranges. Data displaying a non-normal distribution are shown as the median with 25th and 75th percentiles. The correlations between B-cell and plasma Neu1, ST6Gal1, and ST6Gal1/Neu1 levels and disease activity scores, including DAS28-ESR, DAS28-MCP-1, DAS28-CRP, SDAI, and CDAI, were also evaluated. Comparisons between B-cell and plasma Neu1, ST6Gal1, and ST6Gal1/Neu1 levels were made across different disease activity categories in order to fulfill two remission criteria (2005 modified ARA and 2011 ACR/EULAR definitions) [9,10]. All correlations and comparisons were adjusted using Bonferroni correction. ROC curves were used to assess biomarkers for predicting disease activity score subgroups. A *p*-Value of <0.05 was considered statistically significant.

## 5. Conclusions

In this study, the total SIA content of IgG anti-CCP antibodies was inversely correlated with B-cell Neu1 levels. Similarly, plasma Neu1 levels were inversely correlated with SIA/IgG anti-CCP ratios in the DAS28-MCP-1 <2.2 (remission subgroup) but not in the non-remission subgroups. These findings suggest that SIA/IgG anti-CCP ratios are inversely associated with both B-cell and plasma Neu1 levels, particularly in the DAS28-MCP-1-defined remission category, rather than in the DAS29-ESR, DAS28-CRP, SDAI, or CDAI categories. Additionally, SIA/anti-CCP ratios were inversely correlated with plasma Neu1 levels (though this was non-significant after Bonferroni correction) among patients meeting either the 2005 modified ARA remission or the 2011 ACR/EULAR remission criteria.

B-cell ST6Gal1/Neu1 ratios, B-cell Neu1 levels, and/or B-cell ST6Gal1 levels were associated with DAS28-ESR, SDAI, and DAS28-MCP-1 score subgroups, suggesting their involvement in disease activity. These associations were not observed for DAS28-CRP and CDAI measures, with the *p*-Value for B-cell ST6Gal1/Neu1 ratios being non-significant with correlation coefficients higher than −0.200 (Table 3); the reason for this is unclear at present. Future studies should aim to elucidate this point.

Therefore, the five disease activity measures considered (DAS28-ESR, SDAI, CDAI, DAS28-MCP-1, and DAS28-CRP) not only differed in their relationships with immunoregulatory cells, cytokines, and imaging features [21], but also exhibited distinct associations with SIA-related enzymes and anti-CCP sialylation content. In particular, the detection of the plasma ST6Gal enzyme and ST6Gal1/Neu1 ratios offers a new clinically useful indicator for DAS28-MCP-1 remission. In addition, B-cell ST6Gal1/Neu 1 ratios may be used to clinically identify combined remission and low-disease-activity subgroups with DAS28-MCP-1 and SDAI scores.

## Figures and Tables

**Table 1:** Demographic and laboratory data of RA patients.

	Mean ± S.D. or Median	Range or 25th and 75th Percentiles
Gender	F:M = 78:19	Total number = 97
Age	57.5 ± 10.6	32–75
Disease duration (months)	87.4	41.65, 179.15
CRP (mg/L)	1.45	0.78, 3.61
ESR (mm/hr)	13.50	8.00, 26.50
MCP-1 (pg/mL)	95.62	51.42, 151.89
RF (IU/mL)	30.20	11.20, 110.50
Anti-CCP (unit)	183.32	139.54, 244.65
SIA/anti-CCP ratios	0.67	0.06, 1.38
TJC	2	0, 4.5
SJC	1	0, 3.0
DAS28-ESR	3.25 ± 1.40	0–6.62
DAS28-CRP	3.63 ± 1.16	1.40–6.59
SDAI	11.20	2.24, 17.10
CDAI	11.50	2.25, 16.75
DAS28-MCP-1	3.46	2.14, 4.04
HAQ-DI	0	0, 0.5

CRP: C-reactive protein; ESR: erythrocyte sedimentation rate; MCP-1: monocyte chemotactic protein-1; RF: rheumatoid factor; anti-CCP: anti-cyclic citrullinated peptide; SIA/anti-CCP ratios: sialic acid/anti-CCP ratios; TJC: tender joint count; SJC: swollen joint count; DAS28-ESR: disease activity score 28-ESR; DAS28-CRP: disease activity score 28-C-reactive protein; SDAI: Simplified Disease Activity Index; CDAI: Clinical Disease Activity Index; DAS28-MCP-1: disease activity score 28-monocyte chemotactic protein-1; HAQ-DI: Health Assessment Questionnaire-Disability Index. Normal ranges: CRP < 5 mg/L; ESR < 30 mm/h for females and <20 mm/h for males; RF < 15 IU/mL.

**Table 2:** Area-under-curve (AUC) of B-cell enzyme levels against disease activity score subgroups.

Enzymes in Subgroups	AUC	Asymptotic *p*-Values
B-cell ST6Gal1 via SDAI ≤ 11	0.566	0.100
B-cell ST6Gal1/Neu1 ratios via SDAI ≤ 11	0.616	0.004
B-cell Neu1 via SDAI > 3.3	0.541	0.361
B-cell ST6Gal1 via DAS28-MCP-1 ≤ 3.6	0.544	0.301
B-cell ST6Gal1/Neu1 ratios via DAS28-MCP-1 ≤ 3.6	0.600	0.018

Receiver operating characteristic curves were formulated, and AUC with statistical significance was obtained. SDAI: Simplified Disease Activity Index; DAS28-MCP-1: Disease Activity Score 28 with monocyte chemotactic protein-1. SDAI < 11 and DAS28-MCP-1 ≤ 3.6 subgroups belong to the combined remission and lower disease activity subgroup; SDAI > 3.3 indicates the non-remission subgroup.

**Table 3:** Correlation between B-cell ST6Gal1/Neu1 ratios and different DAS28 scores.

	Rho	*p*-Values	Number of Visits
DAS28-ESR	−0.236	<0.001	215
DAS28-CRP	−0.161	0.018	215
SDAI	−0.180	0.008	215
CDAI	−0.187	0.006	217
DAS28-MCP-1	−0.147	0.036	205

DAS28: disease activity score 28; SDAI: Simplified Disease Activity Index; CDAI: Clinical Disease Activity Index; MCP-1: monocyte chemotactic protein-1.

**Table 4:** Area-under-curve (AUC) of plasma enzyme levels against disease activity score subgroups.

Plasma Enzymes in Subgroups	AUC	Asymptotic *p*-Values
Plasma ST6Gal1 via DAS28-ESR < 2.6	0.582	0.046
Plasma ST6Gal1 via DAS28-ESR ≤ 3.2	0.588	0.024
Plasma ST6Gal1/Neu1 ratios via DAS28-ESR ≤ 3.2	0.580	0.039
Plasma ST6Gal1 via CDAI ≤ 2.8	0.593	0.041
Plasma ST6Gal1 via DAS28-MCP-1 < 2.2	0.642	0.001
Plasma ST6Gal1/Neu1 ratios via DAS28-MCP-1 < 2.2	0.628	0.003
Plasma ST6Gal1 via modified 2005 ARA remission	0.619	0.012
Plasma ST6Gal1/Neu1 ratios via modified 2005 ARA remission	0.590	0.056
Plasma ST6Gal1 via 2011 ACR/EULAR remission	0.567	0.152
Plasma ST6Gal1/Neu1 ratios via 2011 ACR/EULAR remission	0.549	0.295

Receiver operating characteristic curves were formulated, and AUC with statistical significance was obtained.DAS28-ESR: disease activity score 28 with erythrocyte sedimentation rate; CDAI: Clinical Disease Activity Index; DAS28-MCP-1: disease activity score 28 with monocyte chemotactic protein-1; ARA: American Rheumatism Association; ACR/EULAR: American College of Rheumatology/European League Against Rheumatism. DAS28-ESR < 2.6: the remission subgroup; DAS28-ESR ≤ 3.2: the combined remission and low-disease-activity subgroup; CDAI ≤ 2.8: the remission subgroup; DAS28-MCP-1 < 2.2: the remission subgroup.

**Table 5:** Multivariate regression analyses for correlations with statistical significance.

Correlation Pairs	Non-Interfering Independent Variables (*p*-Values < 0.05)	Interfering Independent Variables (*p*-Values ≥ 0.05)
Total SIA contents of IgG anti-CCP antibodies vs. B-cell Neu1 levels (Figure 1A)	Age, IgG anti-CCP, HCQ, biologics, CRP, and MCP-1.	Disease duration, MTX, sulfasa, and ESR.
SDAI scores > 3.3 vs. Ln(B-cell Neu1 levels (Figure 2B)	Sulfasa, HCQ, biologics, CRP, and ESR.	Age, disease duration, IgG anti-CCP, MTX, and MCP-1.
SDAI scores ≤ 11 vs. Ln(B-cell ST6Gal1 levels (Figure 2C)	Age, disease duration, IgG anti-CCP, MTX, sulfasa, and HCQ.	Biologics, CRP, ESR, and MCP-1.
SDAI scores ≤ 11 vs. Ln(B-cell ST6Gal1/Neu1 ratios (Figure 2D)	Age, disease duration, IgG anti-CCP, MTX, sulfasa, and HCQ.	Biologics, CRP, ESR, and MCP-1.
DAS28-MCP-1 scores ≤ 3.6 vs. Ln(B-cell ST6Gal1 levels (Figure 2E)	Age, disease duration, IgG anti-CCP, MTX, sulfasa, and HCQ.	Biologics, CRP, ESR, and MCP-1.
DAS28-ESR scores vs. Ln(B-cell ST6Gal1/Neu1 ratios (Table 3)	Age, disease duration, IgG anti-CCP, MTX, sulfasa, and HCQ.	Biologics, CRP, ESR, and MCP-1.
SIA/anti-CCP ratios vs. Ln(plasma Neu1 levels) in the DAS28-MCP-1 scores < 2.2 remission subgroup (Supplementary Figure S1)	Age.	Disease duration, IgG anti-CCP, MTX, sulfasa, HCQ, biologics, CRP, ESR, and MCP-1.

In the correlation pairs column, the first item is the outcome variable. Methotrexate: MTX; sulfasasalazine: sulfasa; hydroxychloroquine: HCQ; SDAI: Simplified Disease Activity Index; Ln: natural logarithm. SDAI < 11 and DAS28-MCP-1 ≤ 3.6 subgroups belong to the combined lower disease activity and remission subgroup; SDAI > 3.3 indicates the non-remission subgroup.

## Data Availability

The data that support the findings of this study are displayed in the article. Others are available from the corresponding author upon reasonable request.

## Supplementary Materials

The following supporting information can be downloaded at: https://www.mdpi.com/article/10.3390/ijms26178226/s1.
