# Supplementary material for: B-Cell ST6Gal1/Neuraminidase 1 Ratios Inversely Predict the Combined Remission and Low-Disease-Activity Subgroup with DAS28-MCP-1 and SDAI Scores for Rheumatoid Arthritis
Source: Int J Mol Sci. 2025 Aug 25;26(17):8226. doi: 10.3390/ijms26178226 (PMC12427851; doi:10.3390/ijms26178226)
Supplement: Supplementary file 1 [file ijms-26-08226-s001.zip › ijms-3733966-supplementary.pdf]

[Supplementary Tables and Figures]

B-cell ST6Gal1/neuraminidase 1 ratios inversely predict the combined remission and low-disease-activity subgroup with DAS28-MCP-1 and SDAI scores for rheumatoid arthritis

Lieh-bang Liou, Ping-Han Tsai, Yao-Fan Fan, Yen-Fu Chen, Che-tzu Chang, Chih-Chieh Chen and Wen-yu Chiang

Supplementary Table S1. Correlations between B-cell SIA-related enzymes, except for Clinical Disease Activity Index categories.

	Number of patients	Correlation coefficients (rho)	*p*-Values
M6.Bneu1.Bratio	123	−0.204	0.024
M6.Bst6.Bratio	123	0.858	< 0.001
M12.Bneu1.Bratio	56	−0.283	0.035
M12.Bst6.Bratio	56	0.841	< 0.001
Bneu1.Bratio.DASESR≧2.6	149	−0.349	< 0.001
Bst6.Bratio.DASESR≧2.6	149	0.737	< 0.001
Bneu1.Bratio.DASCRP≧2.4	173	−0.440	< 0.001
Bst6.Bratio.DASCRP≧2.4	173	0.766	< 0.001
Bneu1.Bratio.SDAI≦3.3	59	−0.266	0.041
Bst6.Bratio.SDAI≦3.3	59	0.809	< 0.001
Bneu1.Bratio.SDAI≧3.3	157	−0.469	< 0.001
Bst6.Bratio.SDAI≧3.3	157	0.753	< 0.001

SIA: α-2,6-sialic acid; M6: month 6; M12: month 12; Bneu1: B-cell neuraminidase 1; Bratio: B-cell ST6Gal1/Neu1 ratios; Bst6: B-cell ST6Gal1, α-2,6-sialyltransferse 1; DASESR and DASCRP: Disease Activity Score-28-ESR and DAS28-CRP; SDAI: Simplified Disease Activity Index.

Supplementary Table S2. Correlations between B-cell SIA-related enzymes across CDAI categories.

	Number of patients	Correlation coefficients (rho)	*p*-Values
Bneu1.Bratio.CDAI (whole)	212	−0.443	< 0.001
Bst6.Bratio.CDAI (whole)	212	0.797	< 0.001
Bneu1.Bratio.CDAI ≦2.8	58	−0.261*	0.048
Bst6.Bratio.CDAI ≦2.8	58	0.805	< 0.001
Bneu1.Bratio.CDAI > 2.8	154	−0.473	< 0.001
Bst6.Bratio.CDAI > 2.8	154	0.733	< 0.001

Bneu1.Bratio.CDAI ≦ 10	102	−0.356	< 0.001
Bst6.Bratio.CDAI ≦ 10	102	0.853	< 0.001
Bneu1.Bratio.CDAI > 10	110	−0.474	< 0.001
Bst6.Bratio. CDAI > 10	173	0.702	< 0.001
Bneu1.Bratio.CDAI ≦22	197	−0.411	< 0.001
Bst6.Bratio.CDAI ≦ 22	197	0.809	< 0.001
Bneu1.Bratio.CDAI > 22	15	−0.636	0.011
Bst6.Bratio.CDAI > 22	15	0.679	0.005
Bneu1.Bratio.CDAI > 2.8 and ≦ 10	44	−0.431	0.003
Bst6.Bratio.CDAI > 2.8 and ≦ 10	44	0.819	< 0.001
Bneu1.Bratio.CDAI > 10 and ≦ 22	95	−0.451	< 0.001
Bst6.Bratio. CDAI > 10 and ≦ 22	95	0.695	< 0.001

SIA: α-2,6-sialic acid; Bneu1: B-cell neuraminidase 1; Bratio: B-cell ST6Gal1/Neu1 ratios; Bst6: B-cell ST6Gal1, α-2,6-sialyltransferse 1; CDAI: Clinical Disease Activity Index. *by Pearson's correlation.

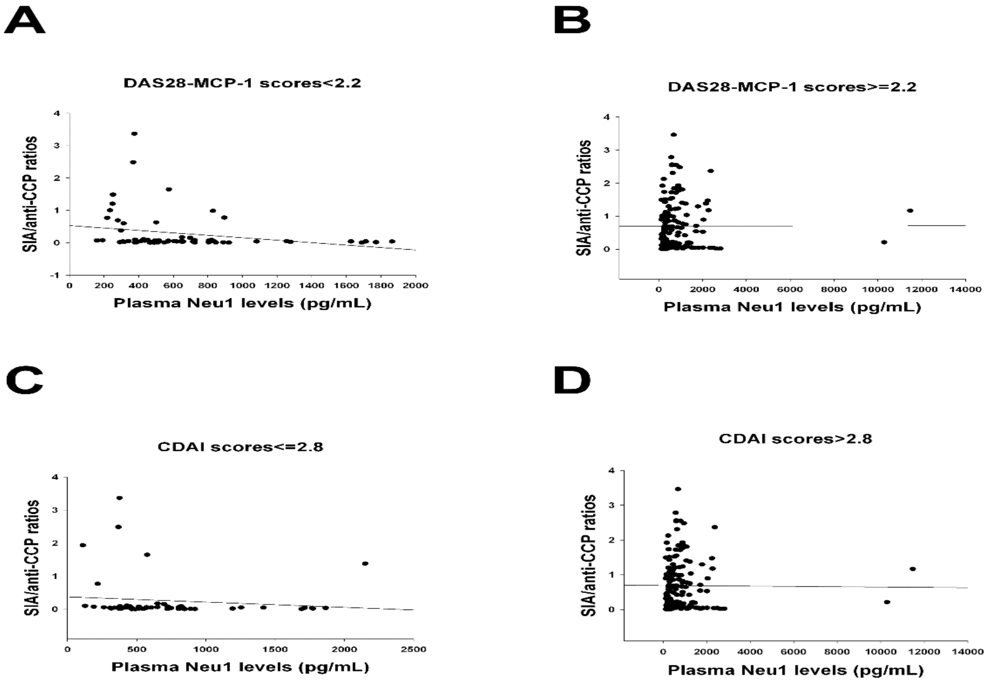

Supplementary Figure S1. Correlation between SIA/anti-CCP ratios and plasma Neu1 levels in remission and nonremission disease activity categories. (A) In patients with DAS28-MCP-1 scores < 2.2 (remission), SIA/anti-CCP ratios were inversely correlated with plasma (free-form) Neu1 levels (n = 64; ϱ = −0.361, p = 0.001). The p-Value is statistically significant for being lower than a significant p-Value = 0.017 (0.05 divided by three correlations). (B) In patients with DAS28-MCP-1 scores ≥ 2.2 (nonremission), no significant correlation was observed (n = 153; ϱ = −0.007, p = 0.933). (C) In patients with Clinical Disease Activity Index (CDAI) scores ≤ 2.8 (remission), SIA/anti-CCP

ratios were inversely correlated with plasma Neu1 levels ($n = 53$; $\rho = -0.289$, $p = 0.036$). The p-Value is statistically non-significant for being higher than a significant p-Value = 0.017 (0.05 divided by three correlations). (D) In patients with CDAI scores > 2.8 (nonremission), no significant correlation was noted ($n = 166$; $\rho = -0.022$, $p = 0.782$). All correlations were assessed using Spearman's correlation analysis.

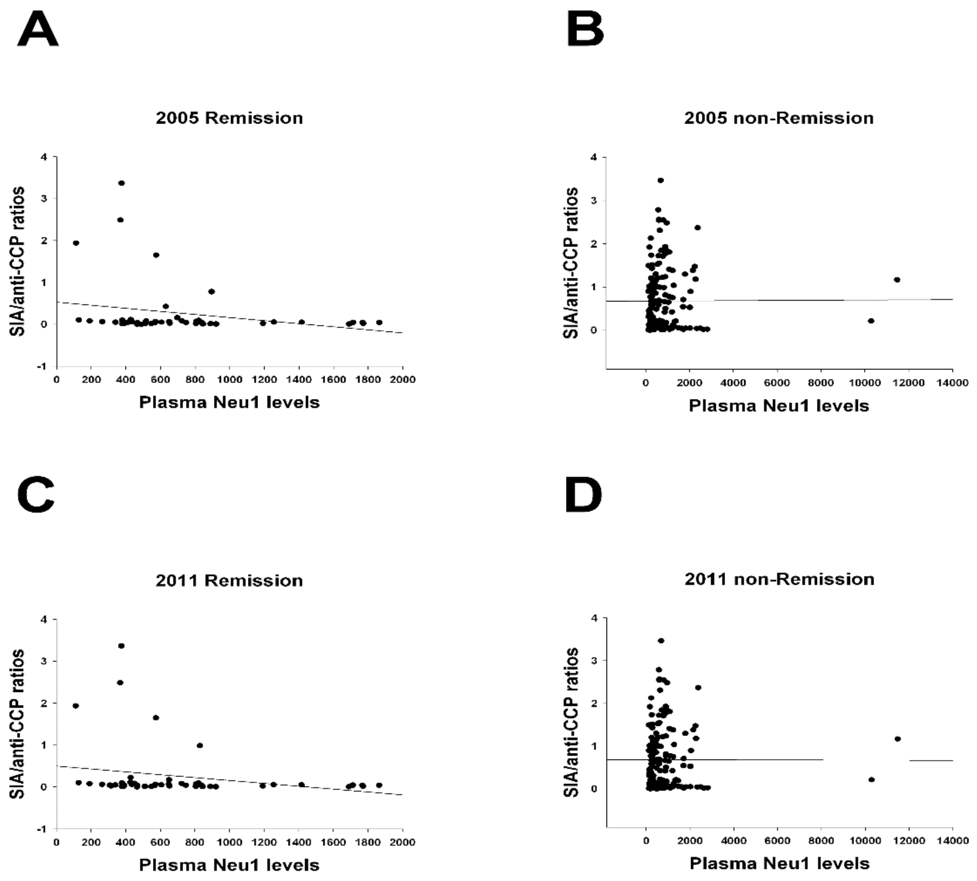

Supplementary Figures S2. Correlations between SIA/anti-CCP ratios and plasma Neu1 levels according to two remission definitions. (A) In patients meeting the 2005 modified American Rheumatism Association (ARA) remission criteria, SIA/anti-CCP ratios were inversely correlated with plasma Neu1 levels ($n = 48$; $\rho = -0.290$, $p = 0.046$). The p-Value is statistically non-significant for being higher than a significant p-Value = 0.017 (0.05 divided by three correlations). (B) No significant correlation was observed in patients not meeting the 2005 modified ARA remission criteria ($n = 168$; $\rho = -0.023$, $p = 0.765$). (C) In patients meeting the 2011 ACR/EULAR remission criteria, SIA/anti-CCP ratios were inversely correlated with plasma Neu1 levels ($n = 50$; $\rho = -0.328$, $p = 0.020$). The p-Value is statistically non-significant for being higher than a significant p-Value = 0.017 (0.05 divided by three correlations). (D) No significant correlation was found in patients not meeting the 2011 ACR/EULAR remission criteria ($n = 167$; $\rho = -0.035$, $p = 0.650$). All correlations were assessed using Spearman's correlation analysis.
